# Microgravity validation of a novel system for RNA isolation and multiplex quantitative real time PCR analysis of gene expression on the International Space Station

**DOI:** 10.1371/journal.pone.0183480

**Published:** 2017-09-06

**Authors:** Macarena Parra, Jimmy Jung, Travis D. Boone, Luan Tran, Elizabeth A. Blaber, Mark Brown, Matthew Chin, Tori Chinn, Jacob Cohen, Robert Doebler, Dzung Hoang, Elizabeth Hyde, Matthew Lera, Louie T. Luzod, Mark Mallinson, Oana Marcu, Youssef Mohamedaly, Antonio J. Ricco, Kathleen Rubins, Gregory D. Sgarlato, Rafael O. Talavera, Peter Tong, Eddie Uribe, Jeffrey Williams, Diana Wu, Rukhsana Yousuf, Charles S. Richey, Julie Schonfeld, Eduardo A. C. Almeida

**Affiliations:** 1 Space Biosciences Research Branch, NASA Ames Research Center, Moffett Field, California, United States of America; 2 Engineering Systems Division, NASA Ames Research Center, Moffett Field, California, United States of America; 3 KBRWyle, Mountain View, California, United States of America; 4 Office of the Director, NASA Ames Research Center, Moffett Field, California, United States of America; 5 Millenium Engineering & Integration Co, Mountain View, California, United States of America; 6 Universities Space Research Association, Mountain View, California, United States of America; 7 Applications Development, Claremont Biosolutions, Upland, California, United States of America; 8 Flight Systems Implementation Branch, NASA Ames Research Center, Moffett Field, California, United States of America; 9 Mission Design Division, NASA Ames Research Center, Moffett Field, California, United States of America; 10 Stanford University, Palo Alto, California, United States of America; 11 NASA Astronaut Corps, NASA Johnson Space Center, Houston, Texas, United States of America; University of Helsinki, FINLAND

## Abstract

The International Space Station (ISS) National Laboratory is dedicated to studying the effects of space on life and physical systems, and to developing new science and technologies for space exploration. A key aspect of achieving these goals is to operate the ISS National Lab more like an Earth-based laboratory, conducting complex end-to-end experimentation, not limited to simple microgravity exposure. Towards that end NASA developed a novel suite of molecular biology laboratory tools, reagents, and methods, named WetLab-2, uniquely designed to operate in microgravity, and to process biological samples for real-time gene expression analysis on-orbit. This includes a novel fluidic RNA Sample Preparation Module and fluid transfer devices, all-in-one lyophilized PCR assays, centrifuge, and a real-time PCR thermal cycler. Here we describe the results from the WetLab-2 validation experiments conducted in microgravity during ISS increment 47/SPX-8. Specifically, quantitative PCR was performed on a concentration series of DNA calibration standards, and Reverse Transcriptase-quantitative PCR was conducted on RNA extracted and purified on-orbit from frozen *Escherichia coli* and mouse liver tissue. Cycle threshold (Ct) values and PCR efficiencies obtained on-orbit from DNA standards were similar to Earth (1 g) controls. Also, on-orbit multiplex analysis of gene expression from bacterial cells and mammalian tissue RNA samples was successfully conducted in about 3 h, with data transmitted within 2 h of experiment completion. Thermal cycling in microgravity resulted in the trapping of gas bubbles inside septa cap assay tubes, causing small but measurable increases in Ct curve noise and variability. Bubble formation was successfully suppressed in a rapid follow-up on-orbit experiment using standard caps to pressurize PCR tubes and reduce gas release during heating cycles. The WetLab-2 facility now provides a novel operational on-orbit research capability for molecular biology and demonstrates the feasibility of more complex wet bench experiments in the ISS National Lab environment.

## Introduction

The ISS National Laboratory is a unique research environment whereby scientists from Academia, Government, and Industry seek to learn the effects of space on life and matter in their many forms. Although a great deal has already been learned about the effects of microgravity on biological systems, [1–3] the large majority of on-orbit experiments do not yield scientific data until long after return of samples to Earth for analysis, creating concerns about timeliness of data availability, and quality of sample preservation [4]. Ground analysis of on-orbit experiments has been necessary because of limited laboratory space, equipment, supplies, and astronaut time resources on ISS, but while this sample return approach maximizes efficient access to space by scientists, it fails to teach us how to conduct end-to-end scientific inquiries in microgravity. As human exploration of the solar system proceeds, human missions to Mars and deep space will require greater levels of autonomy and resourcefulness as outlined in the National Research Council’s Space Exploration Decadal Survey for Life and Physical Sciences [5] An important component of future traveling and living in space away from low Earth orbit will be the ability to perform modern molecular biology analyses, including purifying RNA and conducting Reverse Transcriptase-quantitative PCR (RT-qPCR) or other genomic analyses for disease diagnostics including viral and bacterial infection, environmental microorganism identification and monitoring of food and water, and in any other research application where monitoring gene expression is required.

Although there are increasing numbers of studies of gene expression in microgravity, there are no reports of any methodologies for RNA purification and RT-qPCR under microgravity conditions, effectively preventing on-orbit analysis of gene expression or PCR-based diagnostics from biological samples. Specifically, recent RNA isolation and PCR-based gene expression studies of biological samples on ISS, Space Shuttle and Biosatellites have been conducted on earth after preserved sample return from space [6–14]. Recent efforts by various groups of investigators have sought to develop portions of an overall genomic and molecular biology experimental capability on ISS. Specifically, these include the NASA Genes in Space 1, miniPCR student experiment, [15] and the “Biomolecular Sequencer” Nanopore DNA sequencing demonstration [16, 17] both conducted simultaneously with WetLab-2. Both experiments however, started with genomic DNA samples, not indicative of gene expression, and relied either on sample return to earth for PCR product analysis, (miniPCR), or complex sequencing library preparation on earth prior to nanopore sequencing in space (Biomolecular Sequencer). Neither of these experiments had the end-to-end on-orbit capability to extract RNA from tissue, convert mRNA to cDNA for RT-qPCR, or to generate quantitative gene expression data as we sought to achieve with the WetLab-2 system.

**Fig 1:**
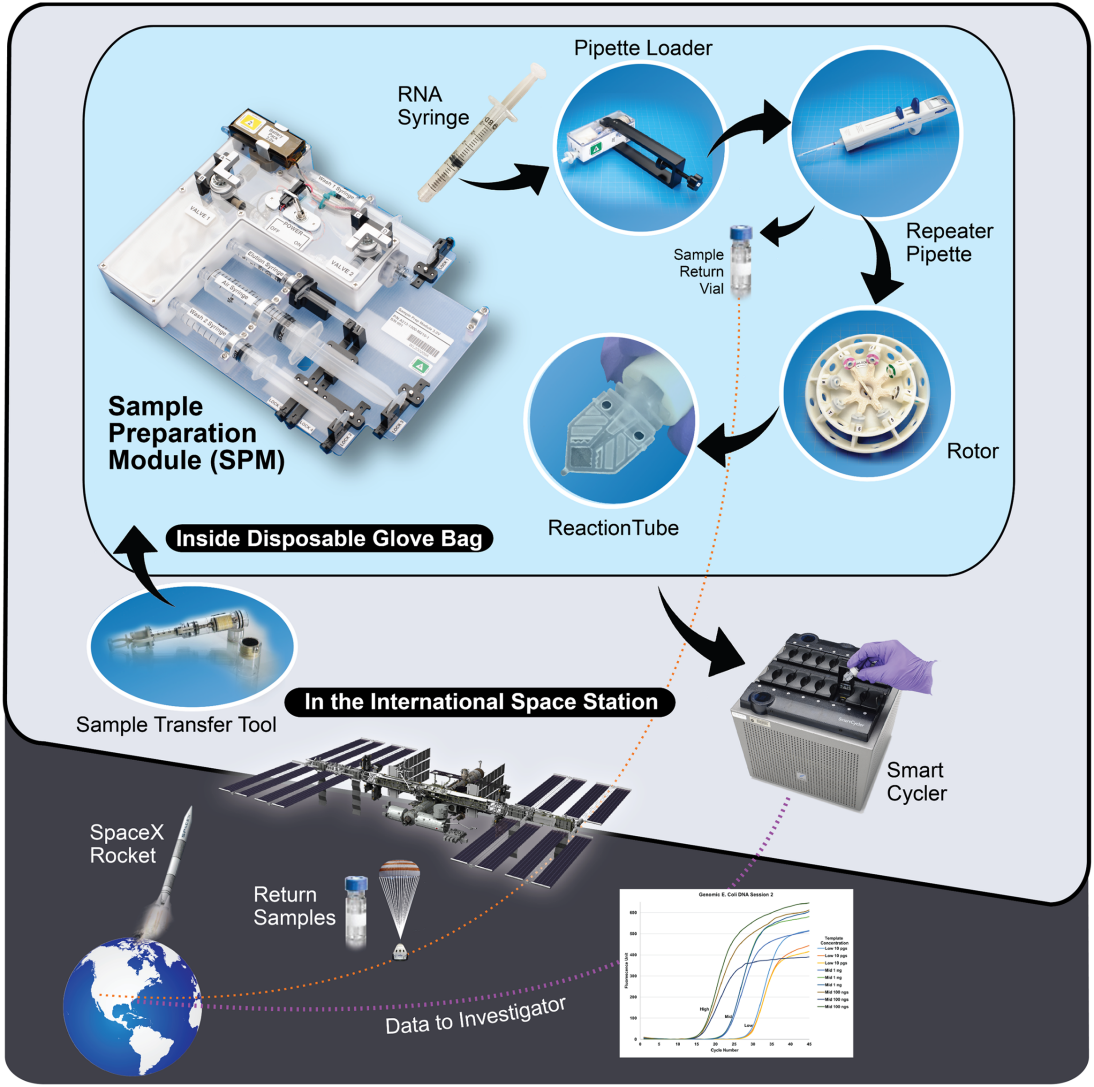
Operations for the WetLab-2 system. Isolation and purification of RNA from biological tissues on–orbit starts with introduction of cells or tissues for lysis and homogenization into the Sample Preparation Module (SPM), followed by RNA binding to an affinity column, washing, and elution from the module. A Pipette Loader (PL) tool is provided for bubble free fluid transfer to a repeater pipette. This is then used to dispense accurate volumes of purified RNA into a centrifuge rotor/rack of lyophilized reagent tubes with enzymes and regents for reverse transcription and Taqman RT-qPCR. Data is available on-orbit within 3h of initiating the experiment, and transmitted by ISS to NASA Marshall Space Flight Center for emailing to investigators within 2 h of clean-up. A more detailed description of the process can be found in the S1 Text.

To address the pressing ISS research and diagnostics need for novel on-orbit end-to-end molecular biology gene expression analysis methods and tools, we devised a robust microgravity fluidic system that can intake diverse biological sample (cells, tissue, surface swabs, blood, etc.) and using temperature-stable reagents, allow for the isolation and purification of nucleic acids, such as RNA, for molecular biology analysis. In addition, the system includes a microgravity compatible thermal cycler, lyophilized reagents and enzymes for PCR assays and methods for reverse transcription of RNA into cDNA, and capabilities for heat inactivation of enzymes such as proteinase K and PCR inhibitors, plus RT-qPCR gene expression analysis using fluorescent TaqMan probes. The WetLab2 suite of instruments and reagents we describe here is novel and unique methodology, substantially distinct from all other existing RNA isolation and RT-qPCR gene expression analysis systems, because of its ability to function in microgravity, with altered fluid flow, hydrostatic pressure, convection, and surface tension conditions of space, while still providing full reagent containment for safe operation on ISS. Using this system, schematically shown in Fig 1, generic reagents and supplies can be prepositioned on ISS to flexibly support science opportunities such as optimizing plant growth, monitoring the evolution of bacterial communities in biofilms, or analyzing gene expression in immune cells from blood, among many other applications. In addition, the WetLab-2 system was also designed to be capable of purifying RNAs for return to Earth, or for other on-orbit molecular biology analyses such as nanopore array-based mRNA sequencing studies, greatly increasing the analysis capabilities of the system and possibilities for discovery in space.

In its validation flight, WetLab-2 successfully demonstrated its capabilities to conduct molecular biology experimentation in microgravity. This consisted of first demonstrating that qPCR functions in microgravity conditions, followed by demonstration of RNA isolation from bacterial cell and mammalian tissue and gene expression analysis by RT-qPCR. When gases evolved by thermal cycling accumulated in PCR tubes due to microgravity conditions interfered with data uniformity, the system demonstrated its flexibility and adaptability to on-orbit experiment modification and iteration in real-time. WetLab-2 also used volunteer astronaut time and spare supplies to design and execute follow-up experiments while on-orbit to resolve the gas bubble problem. Below we describe in detail the design and scientific validation of the WetLab-2 suite of methods, reagents, and hardware for RNA purification and gene expression analysis by RT-qPCR, enabling future molecular biology experimentation to be conducted end-to-end in real-time on ISS, and allowing data interpretation and experiment redesign while on-orbit.

## Materials and methods

In order to validate the system, a number of ground-based and on-orbit tests were performed including: validation of *E*. *coli* primer assays (ground), validation of lyophilized components (ground), validation of the SPM (ground), validation of all hardware (ground), validation of qPCR efficiency using pre-prepared assays (on-orbit), validation of RNA extracted from *E*. *coli* (on-orbit), and from mouse liver tissue (on-orbit) using the SPM. The methods used in these validation experiments are described in detail below.

### Primer and probe design

This study used three types of assays for different portions of the validation of the system. First, an *E*. *coli* genomic assay was used to demonstrate if qPCR was possible in microgravity and that the data matched 1 g controls. This was followed with a set of *E*. *coli* gene expression assays designed to validate the sample prep and RT-qPCR capabilities of the system. Finally, a set of mouse gene expression assays were used to validate the system with a mammalian tissue.

The *E*. *coli* primers and probes used in this study were designed in-house to target both genomic and exonic regions. The *E*. *coli* genomic assay consisted of primers targeting the 16S ribosomal DNA sequence for *rrnH* gene while the gene expression assays targeted the following genes: heat shock protein 70–2 (*dnaK*), RNA polymerase (*rpoA*), and a glucitol operon repressor (*srlR*). For mouse assays, pre-designed intron spanning assays targeting genes for glyceraldehyde 3-phosphate dehydrogenase (gapdh) and 60S ribosomal protein L19 (rpl19) were used for normalization and fibronectin 1 (fn1) was used to measure gene expression analysis. All primers and probes were produced by Integrated DNA Technologies (IDT, Coralville, Iowa). In all cases, TaqMan based technology was used [18]. The corresponding fluorescent dye and its primer and probe sequences are shown in Table 1.

**Table 1:** *E*. *coli* and mouse liver primers and probes used in this study.

Organism/ Assay type	Gene Name	Primer/Probe	Sequence
*E*. *coli /* genomic	16S genomic region	Probe	/56-FAM/ AC GTT TGA C/ZEN/C TCG TCT TTC GGT GTG T/3IABKFQ/
		Forward	GCC AAG ACC AAC CAA TGA CAG CAA
		Reverse	ACC TGC TCT CTT CGC GTA CTG TTT
*E*. *coli /* gene expression	*dnaK*	Probe	/56-FAM/ TGC TGA GTT /ZEN/ CTT TGG TAA AGA GCC GCG T/3BHQ_1/
		Forward	TGG TGG TCA GAC TCG TAT GCC AAT
		Reverse	ATT GCT ACA GCT TCG TCC GGG TTA
	*rpoA*	Probe	/5HEX/ TTC GTC GTG /ZEN/ CGG CAA CCA TT/3IABkFQ/
		Forward	TAG AAC AGC GTA CCG ACC TG
		Reverse	CGA AAG CTT CCA GTT GTT CA
	*srlR*	Probe	/5Cy5/ CCA CAG GCA CAA CCA TTC GCA /3IAbRQSp/
		Forward	ATT GGC GCA ATA CTT TGA CA
		Reverse	GCA TGT TCC AGA ATG ACC AG
mouse liver */* gene expression	gapdh	Probe	/56-FAM/ TGC AAA TGG /ZEN/ CAG CCC TGG TG/3IABkFQ/
		Forward	GTG GAG TCA TAC TGG AAC ATG TAG
		Reverse	AAT GGT GAA GGT CGG TGT G
	rpl19	Probe	/5HEX/ TGA CCG CCA/Zen/T ATG TAT CAC AGC CTG /3IABkFQ/
		Forward	GAT GCG CTT GTT TTT GAA CAC
		Reverse	CCG GCT TCT CAG GAG ATA C
	fn1	Probe	/5Cy5/ CTG ATC GTT GCA TCT GTT TCT GGA GGT/3IAbRQSp/
		Forward	GTT TCC TCG GTT GTC CTT CT
		Reverse	GAC TGT ACT TGT CTA GGC GAA G

*E*. *coli* primers and probes were designed in-house, mouse assays were selected from those available from IDT. 56-FAM: 5' 6-FAM (Fluorescein), ZEN tm IDT Trademark dark quencher, 3IABKFQ: 3’ Iowa Black Fluorescent Quencher, 3BHQ_1: 3’ Black Hole Quencher, 5HEX: 5’ Hexachlorofluorescein, 5Cy5: 5’ Cyanine5; 3IAbRQSp: 3' Iowa Black^®^ RQ-Sp

### Assay formulation, preparation, and validation

To validate the *E*. *coli* genomic assay formulation, we analyzed qPCR performance using varying quantities of template in tenfold dilutions between 1 μg and 1 pg (inclusive). Assays were constituted using high molecular weight genomic DNA from *E*. *coli* type B cells (Affymetrix, Sunnyvale, CA), custom primers and probe targeting a 16S ribosomal DNA sequence (IDT, Coralville, Iowa), GoTaq Probe Master Mix (Promega, Madison, WI), and molecular biology grade water (TekNova, Hollister, CA). Amplification was achieved via a standard thermal protocol that included a hot-start activation step (95°C, 2 min) and 45 cycles of denaturation (95°C, 15 s) and annealing/extension (54°C, 60 s). Samples were run in duplicate technical replicates and their average Ct values were plotted against template concentration to determine R-square and efficiency of amplification. Products were confirmed using a Bioanalyzer 2100 and an Agilent DNA chip, using a DNA 1000 protocol and reagent.

Genomic and gene expression assays for *E*. *coli* (*dnaK*, *rpoA* and *srIR*) and mouse tissue (gapdh, rpl19, and fn1) were validated using genomic *E*. *coli* DNA and RNA from *E*. *coli* K12 TB1 (New England Biolabs Inc, Ipswich, MA) and whole livers from male C57BL/6 12–16 week perfused/snap frozen (Charles River, Wilmington, MA). Initial feasibility studies however utilized mouse liver tissues obtained under NASA Ames Research Center IACUC protocols NAS-14-002-Yr1-3 and NAS-15-008-Y1-2 (E. Almeida and E. Blaber). RNA was isolated using the RNeasy Mini kit (Qiagen, Valencia, CA) and tested to have a RNA Integrity Number (RIN) greater than 9.0 using a Bioanalyzer 2100 and RNA 6000 Pico Chip. Each assay was validated using the appropriate RNA template in a RT-qPCR cocktail. Singleplex, duplex and triplex reactions were validated for each organism. Amplification was achieved via a single step RT-qPCR thermal protocol that includes a Reverse Transcription (45°C, 15 min), hot-start activation step (95°C, 2 min) and 45 cycles of denaturation (95°C, 15 s) and annealing/extension (54°C, 60 s), further detailed online at protocols.io [19].

### Lyophilized assay validation

After successful validation of assay performance, all assay formulations were purchased as lyophilized sample-ready products in the Reaction Tubes (BioGX, Birmingham, AL). The protocol for lyophilized assay generation is detailed in online at protocols.io [20]. To test the performance of the lyophilized assays, Ct values of lyophilized assays were compared to those of non-lyophilized controls. For the *E*. *coli* genomic assay three concentrations of template; high (100 ng), mid (1 ng), and low (10 pg), along with no template controls (NTC), were run for each group. Non-lyophilized controls were constituted as described above while lyophilized assays were re-hydrated with elution buffer (Claremont BioSolutions, Upland, CA). Lyophilized *E*. *coli* and mouse liver gene expression assays were tested by running singleplex, duplex, and triplex reactions. Individual, average, and standard deviation of Ct values were compared to corresponding controls, within template groups. Two individual lots were tested against a single benchtop control lot. BioGX also carried out independent validation of the lyophilized products.

### Microgravity compatible RNA extraction procedure

RNA isolation was performed utilizing an alcohol-free version of the RNA binding buffer and washes for Claremont BioSolutions’ commercially available OmniLyse^®^ [21, 22] and their RNAexpress^™^ column. The process was carried out in a SPM, an enclosed microgravity-compatible hardware module designed to require only simple manual manipulations that can be performed by an untrained user. The SPM contains syringes preloaded with wash and elution buffers and has an input port with a Luer-Lok^®^ connection for the incoming Sample Transfer Tool (STT). The procedure consists of lysing the sample with the battery powered OmniLyse^®^ and binding of RNA to the RNAexpress^™^ column. This is followed by two washes of the column with Claremont BioSolutions wash buffer in preloaded syringes. The RNA is then eluted into a removable syringe using the Claremont BioSolutions elution buffer.

To test the SPM performance, 100 million *E*. *coli* cells or 5 mg of mouse liver tissue were used for RNA extraction. Resulting RNA quantity and quality were assessed using a NanoDrop 2000^®^ (Thermofisher, Waltham, MD) and a Bioanalyzer 2100 (Agilent, Santa Clara, CA), respectively. The SPM-derived RNA was also used as the template in RT-qPCR reactions prepared as stated above.

### Hardware testing

To determine the compatibility of hardware components, reagents were stored in hardware for long duration testing and were assessed for compatibility with downstream molecular biology applications (e.g. qPCR, RNA Isolation, and RT-qPCR).

ACT^2^s (specialty microgravity compatible transfer hardware, Techshot, Greenville, IN) and Finger Loop syringes (BD, Franklin Lakes, NJ) were loaded and stored at -80°C. Units were removed from the freezer at designated time points, allowed to thaw at ambient temperature (at least 1 h for the ACT^2^ and 45 min for the Finger Loop syringe), then RNA was extracted using the SPM procedure described above. To test the SPMs for reagent compatibility, SPMs were assembled with the syringes preloaded with wash and elution buffers and stored at ambient temperatures. At designated time points, SPMs were used to isolate fresh samples of either 1x10^8^ *E*. *coli* cells or 5 mg of mouse liver tissue. For all hardware tests, RNA quantity and quality were assessed as described previously. RT-qPCR using sample specific primers and probe were also used to test RNA quality as described previously.

Reaction Tubes containing lyophilized *E*. *coli* and mouse gene expression assays, master mixes and enzymes were packaged at BioGX (8 tubes per sealed pouch) and stored at 4°C until tested. All lyophilized mixes were tested at designated time points and utilized 25 ng of *E*. *coli* or mouse Qiagen RNeasy purified RNA (as appropriate) in 25 μl of molecular biology grade water to rehydrate the assays. Amplification conditions used were described above. In all cases, results were compared to those from the t = 0 time point. The thermocycler to be flown to the ISS was also validated prior to use.

Details of all hardware testing results are shown in the S2 Text.

### On-orbit quantitative PCR analyses

Microgravity data generated in this study were attained from on-orbit operations onboard the ISS. All materials, hardware, and biology were transported during the SpaceX CRS-8 cargo resupply mission that launched to the ISS on April 8^th^, 2016 and experiments were conducted on April 19^th^, 22^nd^, 26^th^, 29^th^ and May 2^nd^. Operations required the use of the WetLab-2 hardware suite consisting of microgravity-compatible STT (ACT^2^ or Finger Loop syringe), SPM, bubble-removing Pipette Loader (PL), reaction tube centrifugation rotor and a Cepheid SmartCycler^®^ for thermocycling/fluorescence readout, as described in S1 Text. 1 g control data were generated using identical protocols and hardware within 12 h of completion of ISS operations. The on-orbit operations consisted of three types of tests. First, the efficiency of qPCR in microgravity using pre-prepared reaction mixtures including *E*. *coli* genomic DNA was tested three times. Second, isolation of RNA on-orbit using the SPM and pre-frozen *E*. *coli* cells followed by analysis of gene expression was tested followed by testing of RNA isolation from mammalian tissue using the SPM and pre-frozen mouse liver tissue followed by analysis of gene expression.

For each experiment, one ISS and one ground kit were removed from their respective cold stowage (both 4°C and -80°C if applicable) and allowed to equilibrate at room temperature for 2–7 h. For ISS operations, experiments were performed within a RNase-free and cleaned Disposable Glove Bag (DGB) to prevent contamination. At the start of ISS operations, session materials contained in RNase-free bags were placed into the DGB and wiped with RNase AWAY^™^ wipes (Molecular BioProducts^™^, Waltham, MA). The 1 g control experiment was conducted in a standard molecular biology laboratory; all work surfaces were cleaned using RNase AWAY^™^ wipes and 70% ethanol. Data from the ISS experiments were downlinked within 2 h of completion of the clean-up activity and results were statistically compared with those from corresponding 1 g controls. A total of five ISS runs were performed; one dataset was lost due to a PL hardware malfunction. All five 1 g control experiments were performed nominally.

The efficiency of qPCR performed in microgravity was determined using reaction mixtures containing low, mid, or high concentrations of *E*. *coli* DNA template and a no template control (NTC) as described above. Reagents were lyophilized in custom capped SmartCycler^®^ reaction tubes on December 1^st^, 2015. Four tubes of each concentration were packaged into tube kits. All tube kits and the elution buffer were transported and stored at 4°C to minimize loss of enzyme activity and maintain quality. On-orbit operations consisted of de-bubbling the buffer using the PL and loading it into a repeater pipette that was used to add 25 μl to each reaction tube. The tubes were centrifuged and loaded into the SmartCycler to analyze qPCR efficiency.

RNA isolation using the SPM in microgravity was validated using 1x10^8^ *E*. *coli* cells grown on the ground and transferred into a STT (ACT^2^) and stored at -80°C or colder. *E*. *coli* RNA isolation and RT-qPCR was performed on April 29^th^ 2016. Tube kits for this study consisted of four tubes each of singleplex assay dnaK-FAM, singleplex rpoA-HEX, duplex (dnaK-FAM and rpoA-HEX), and triplex (dnaK-FAM, rpoA-HEX and srlR-Cy5). All assay and RT-qPCR reagents were lyophilized in SmartCycler^®^ Reaction Tubes on Dec 1^st^, 2015. The tube kits were transported and stored at 4°C until used while other hardware and consumables were kept at ambient. On-orbit operations consisted of processing the bacterial culture through the SPM, followed by de-bubbling and tube loading as stated above. The tubes were centrifuged and loaded into the SmartCycler^®^ for gene expression analysis with RT-qPCR.

To test isolation of RNA from mammalian tissue in microgravity using the SPM, 5 mg of mouse liver tissue were biopsied on the ground and transferred into a STT (10 ml finger loop syringe) with 2 ml of lysis buffer (Claremont BioSolutions, Upland, CA). Samples were flash-frozen in liquid nitrogen and stored at -80°C on March 4^th^, 2016. Mouse liver tissue RNA isolation and RT-qPCR was performed on May 2^nd^ 2016. Tube kits for this study consisted of four tubes each of singleplex assay gapdh-FAM, singleplex rpl19-HEX, duplex (gapdh-FAM and rpl19-HEX), and triplex (gapdh-FAM, rpl19-HEX and fn1-Cy5). The reagents were lyophilized and transported as described above. On-orbit operations were the same as those for the bacterial culture.

A follow on experiment was performed on October 19^th^, 2016 during increment 49, using a spare *E*. *coli* kit. This experiment consisted of a repeat of the *E*. *coli* gene expression study, however, at the start of operations, modified septa caps were removed from 4 technical replicates of the singleplex reaction (dnaK-FAM) and 4 technical replicates of the duplex reaction (dnaK-FAM, rpoA-HEX) tubes. After addition of the RNA, the reaction tubes were closed using clipped tube caps from commercial Cepheid SmartTubes (Cepheid, Sunnyvale, CA). The 1 g control experiment was conducted with a 24 h delay.

### Analyses and statistics

Cepheid SmartCycler 2.0d software was used to analyze all fluorescence intensities and generate Ct values. All pre-launch ground testing used the default software values (Background Subtraction: ON, Background Min: 5, Background Max: 40, Primary Curve Analysis, Manual Threshold: 30, Boxcar Averaging: 0), while microgravity to 1 g comparisons used slightly modified values with a Boxcar averaging of 3. Genomic DNA qPCR runs used: Background Subtraction: ON, Background Min: 5, Background Max: 40, Primary Curve Analysis, Manual Threshold: 30, Boxcar Averaging: 3; software values for the *E*. *coli* RT-qPCR runs: Background Subtraction: ON, Background Min: 1, Background Max: 40, Primary Curve Analysis, Manual Thresholds: (FAM: 15, HEX: 15, Cy5: 15) Boxcar Averaging: 3; and software values for the mouse liver RT-qPCR runs: Background Subtraction: ON, Background Min: 5, Background Max: 40, Primary Curve Analysis, Manual Thresholds: (FAM: 15, HEX: 30, Cy5: 15) Boxcar Averaging: 3; for the Increment 49 Bubble Mitigation run: Background Subtraction: ON, Background Min: 1, Background Max: 40, Primary Curve Analysis, Manual Thresholds: (FAM: 15, HEX: 15, Cy5: 15) Boxcar Averaging: 0. Amplification efficiency was calculated using the formula: E = (10^[–1/slope]^-1)*100.

Statistical analyses were performed using JMP10 (SAAS Institute Inc, Cary, NC) and GraphPad Prism 5 (GraphPad Software Inc, La Jolla, CA). Comparisons made between microgravity and 1 g control data, or between lyophilized and benchtop control assays across different template concentrations were evaluated using ANOVA or Student’s *t-test* as appropriate. In all cases, figures report the mean ± standard deviation (SD) and *p* ≤ 0.05 was considered the threshold for significance. Outliers were calculated using Grubb’s test with alpha = 0.05.

## Results

### qPCR in microgravity

#### Assay design and testing

In order to assess the functionality of PCR in microgravity conditions and to establish the dynamic range and efficiency of amplification, we designed a robust quality control qPCR assay to measure these parameters. We targeted a 16S ribosomal region of genomic *E*. *coli* DNA. The abundant target expression improved precision, sensitivity, and reproducibility, providing robust assay conditions.

**Fig 2:**
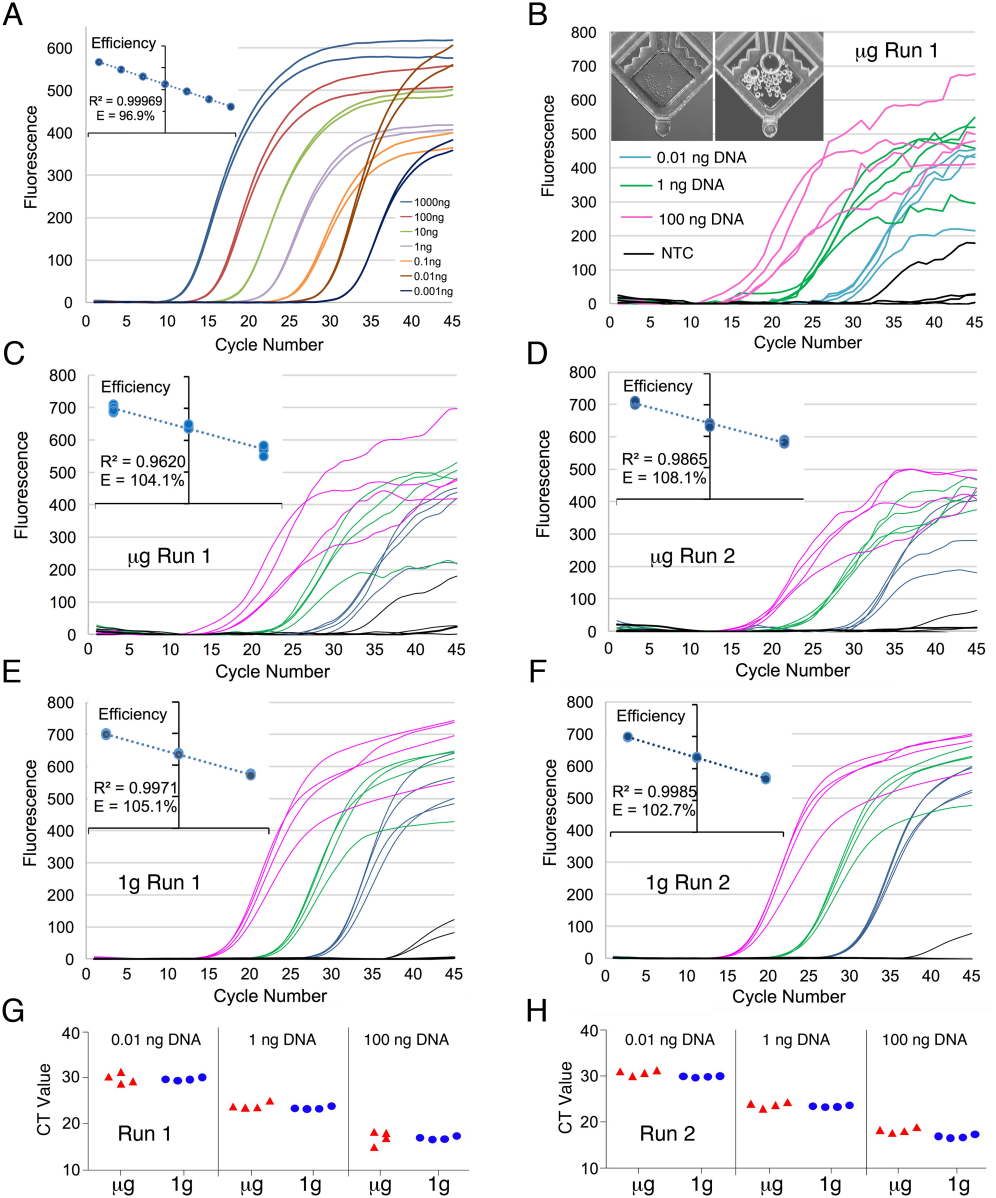
qPCR validation in microgravity. (A) qPCR assays targeting the *E*. *coli* 16S ribosomal gene were validated on the ground using seven tenfold dilutions of template to measure sensitivity and efficiency (inset). (B) Amplification curve from qPCR run conducted on the ISS with photo of a representative tube pre- and post- run in microgravity (inset). (C-F) qPCR amplification curves from the microgravity (C & D) and 1 g control (E & F) runs. All curves use the default SmartCycler values with the exception that the boxcar correction is set to 3. Efficiency graphs are shown on the insets. (G & H) Ct values for low (0.01 ng/test), mid (1 ng/test), and high (100 ng/test) assays during both experimental runs are shown using scatter plots with jitter. μg = microgravity.

Validation of the *E*. *coli* genomic DNA assay was performed using a standard thermal cycling protocol with controlled quantities of commercially available *E*. *coli* DNA as template. Assays were tested with template quantities between 1 pg and 1 μg, in duplicates (Fig 2A), and evaluated based on amplification plot characteristics and reaction efficiency. Results show that assays successfully generated amplification products, as detected by qPCR, with fluorescence peaks between 300 and 600 units. Assay efficiency was determined to be at 96.8% with an R^2^ greater than 0.99 (Fig 2A inset). Specificity and accuracy was confirmed by gel band migration analysis, with the product amplicon generating bands of the expected molecular size.

In order to minimize crew time and the need for refrigeration resources, pre-formulated reagents were lyophilized in the Cepheid SmartTube detection window. To validate that the lyophilized assay format and lyophilization process did not compromise assay performance [23], we tested the lyophilized products by assessing qPCR performance in comparison to commercial non-lyophilized reagents (control). This was performed using high, mid and low template concentrations and a NTC. Combined data between two lots of lyophilized product show that lyophilized assays were able to consistently generate Ct values similar to those generated by non-lyophilized controls (see Table 2).

**Table 2:** Validation of lyophilized assays.

**Genomic *E*. *coli* DNA Assay**	**High DNA Template**	**Medium DNA Template**	**Low DNA Template**
Control	15.71 +/- 0.16	23.55 +/- 1.5	29.14 +/- 0.15
Lyophilized	16.43 +/- 0.51	22.87 +/- 0.43	29.87 +/- 0.32
***E*. *coli* RNA Assay**	**dnaK-FAM Ct**	**rpoA-HEX Ct**	**srlR-Cy5 Ct**
Control	25.79 +/- 0.31	24.4 +/- 0.11	29.32 +/- 0.33
Lyophilized	24.99 +/- 1.52	23.27 +/-0.16	28.53 +/- 0.56
**Mouse Liver RNA Assay**	**gapdh-FAM Ct**	**rpl19-HEX Ct**	**fn1-Cy5 Ct**
Control	21.11 +/- 0.085	19.35 +/- 0.092	21.57 +/- 0.092
Lyophilized	21.59 +/- 0.41	19.33 +/- 0.33	21.17 +/- 0.085

Lyophilized and non-lyophilized assay comparison of qPCR cycle threshold data for *E*. *coli* DNA Assay for (High, Mid, Low) DNA template amount, and RT-qPCR cycle threshold data for *E*. *coli* and mouse liver RNA. There are no statistical differences between lyophilized assays and commercial non-lyophilized assays. Data shows Ct values +/- SD.

#### Microgravity qPCR runs

Following transport to the ISS and an initial software and instrument setup, the system was tested with three runs of the *E*. *coli* genomic DNA assay. Data from the first run is shown in Fig 2B and has a typical qPCR amplification curve, although an unusual amount of noise was observed in microgravity samples especially in later cycles. Furthermore, when the default SmartCycler software values were used, outliers were observed resulting in high standard deviations between replicate samples. These outliers did not impair the clustering of the on-orbit data with the respective DNA concentrations.

Data from a second run was similar to the first but a third repeat run did not yield results due to tube dispensing errors attributed to a PL malfunction. Post-run photos of the tubes showed that no liquid was transferred into most of the tubes.

Noise in the curves was attributed to degassing bubble formation in the tube detection windows during thermal cycling [24–26], as confirmed by post-run photos of the tubes. Photos of all tubes were taken before and after thermal protocol completion, the inset in Fig 2B shows pre- and post-run photos of a representative tube for reference. The pre and post photos of the tubes reveal significant bubble formation and microgravity-trapping during the heating cycles of the qPCR reaction. The bubbles were likely caused by degassing during thermocycling [26] and their evolution and trapping may cause noise in fluorescence intensity detection. We used the SmartCycler software boxcar averaging to smooth out the amplification curve noise, but otherwise kept the default software values. All statistical comparisons for this study used this data.

#### Microgravity and 1 g control data comparisons

Shortly after the completion of each of the on-orbit operations, a 1 g control run was performed. Care was taken to match the removal time of the lyophilized assays from 4°C to the start of operations. Fig 2C–2F shows side-by-side comparisons of microgravity runs and their matched 1 g controls. Amplification curves from the corresponding 1 g control runs were smooth and similar to those obtained during previous testing. Non-specific amplification, as observed from the NTC tubes (black lines), were seen on both microgravity and 1 g control graphs. Given that the target gene is a 16S rRNA, non-specific amplification in the NTC samples may be seen when Taq polymerase is bacteria-derived [27] or due to primer derived artifacts. Microgravity fluorescence intensity values also show a lower overall amplitude potentially due to bubbles blocking/reducing some of the collected fluorescence signal [24–26]. Raw background fluorescence data between microgravity and 1 g control were also compared and details can be found in the S1 File.

Comparison of microgravity and 1 g control Ct values revealed that despite the noise and reduced intensity in microgravity samples, the Ct values still appear to be comparable to 1 g control results as seen in Fig 2G & 2H and in S1 Table.

Efficiency plots for the microgravity and 1 g control samples (Fig 2C–2F insets) were generated with Ct values using a Boxcar averaging of 3 and otherwise default software settings. The efficiency for the 1 g control data was 105.1% for run 1 and 102.7% for run 2 and the linear standard curve R^2^ = 0.9971 and 0.9985 respectively and microgravity data was 104.1% and 108.1% and R^2^ = 0.9620 and 0.9865 respectively. One microgravity PCR efficiency was slightly outside of the optimal efficiency range of 90–105%, likely due to the effects of bubbles present in the detection window.

### RNA sample prep and RT-qPCR analysis in microgravity

#### Assay design and testing

*E*. *coli* and mouse gene expression assays were tested and validated using Qiagen RNeasy derived RNA. For both species, genes were tested using the same singleplex, duplex, and triplex formats that were planned to be used on ISS. Table 3 shows representative Ct value data. All primer and probe sets yielded consistent RT-qPCR reactions with resulting Ct values between 19 and 24 when 100 ng of starting RNA was used.

**Table 3:** *E*. *coli* and mouse liver assay validation using 100 ng control RNA.

***E*. *coli* RNA**	**dnaK-FAM**	**rpoA-HEX**	**srlR-Cy5**
Singleplex	19.8 +/- 0.53		
Singleplex		19.6 +/-0.40	
Duplex	20.4 +/- 0.80	20.3 +/- 0.39	
Triplex	19.6 +/- 0.91	20.3 +/- 0.47	26.8 +/- 0.78
***Mouse Liver* RNA**	**gapdh-FAM**	**rpl19-HEX**	**fn1-Cy5**
Singleplex	21.5 +/- 0.24		
Singleplex		21.2 +/- 0.19	
Duplex	22.5 +/- 0.41	21.3 +/- 0.35	
Triplex	23.9 +/- 0.43	23.1 +/- 0.36	25.8 +/- 0.44

Lyophilized *E*. *coli* and mouse RNA gene expression assays, singleplex, duplex and triplex formulations, were tested as described above. All lyophilized assays generated similar PCR amplification compared to the non-lyophilized controls indicating that the more labile Reverse Transcriptase enzyme [28, 29] remained active. Table 3 shows the results from the triplex assays, results generated by the singleplex and duplex assays yielded similar results. ANOVA analyses of these results showed no statistical variation between lyophilized assays and their non-lyophilized controls, therefore indicating that lyophilized assays were suitable for use on ISS.

#### SPM design and testing

**Fig 3:**
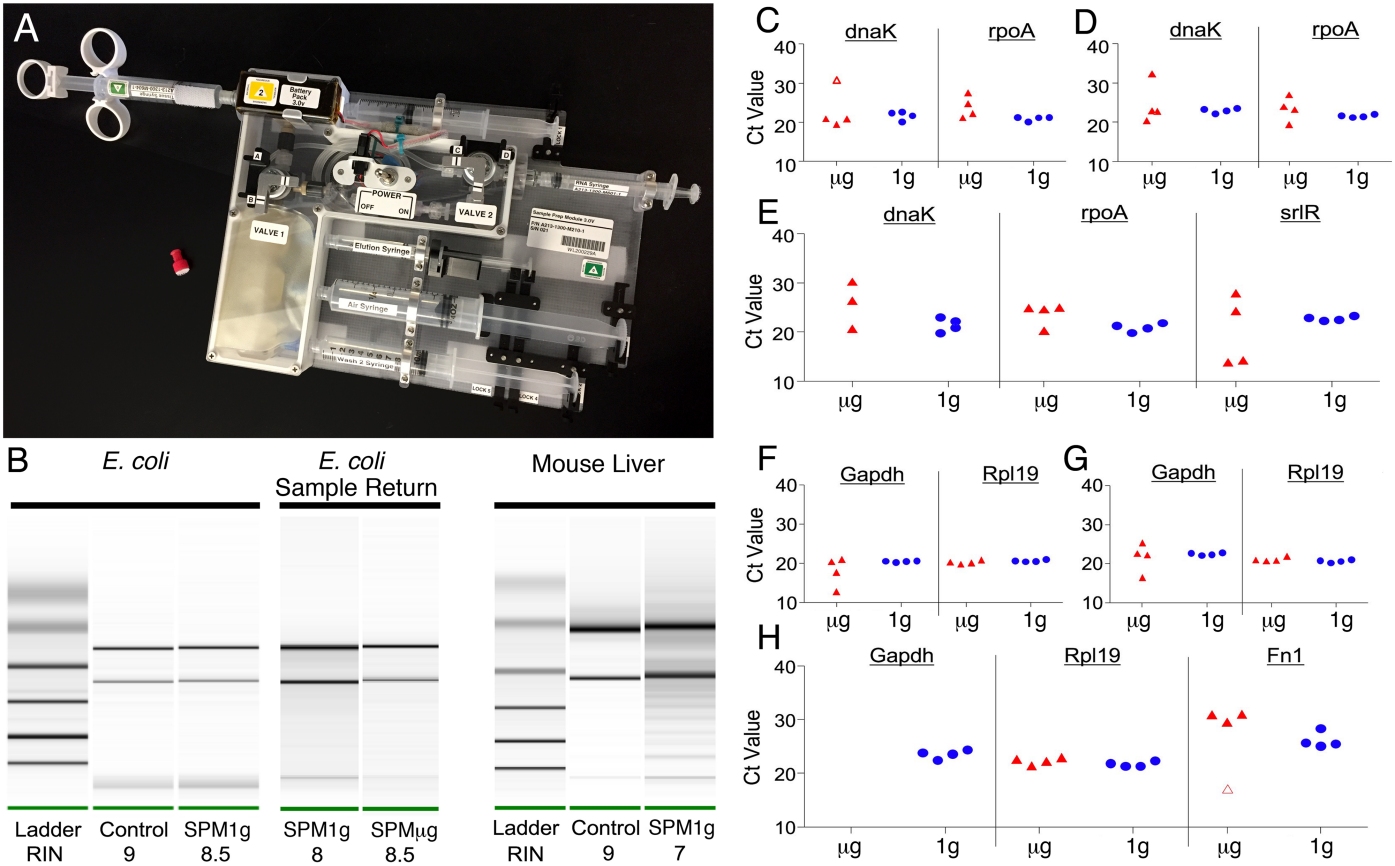
RNA isolation and RT-qPCR in microgravity. (A) Photo of SPM. (B) Typical RNA quality from SPM with *E*. *coli* (left panel) and mouse liver (right panel), control Qiagen (left lane) SPM (right lane). Center panel shows RNA quality from the 1 g control (left lane) and the returned microgravity sample from ISS (right lane). (C-E) Scatter plots with jitter of the microgravity and 1 g control *E*. *coli* singleplex (C), duplex (D) and triplex (E) reactions. One outlier is indicated by the open marker in C. One of the microgravity triplex tubes did not give a dnaK-FAM signal (E). (F-H) Scatter plots with jitter of the microgravity and 1 g control mouse liver singleplex (F), duplex (G) and triplex (H) reactions. One outlier from the microgravity triplex fn1 plot is indicated by the open marker and no gapdh-FAM signal was seen in the microgravity triplex reactions (H).

An enclosed, simple RNA extraction procedure not requiring toxic chemicalsthat may complicate operation on ISS, was adapted (by removing alcohol) from commercially available Claremont BioSolutions products (Fig 3A). The resulting SPM was tested to ensure that the RNA yield was of sufficient quantity and quality for RT-qPCR [30]. Following optimization, RNA yields from the SPM were 2–10 μg for 1 x 10^8^ *E*. *coli* and 1.5–8 μg for 5 mg mouse liver. The RNA quality was adequate with RINs of 7.9 ±1.2 for *E*. *coli* and 7.4 ±1.6 for mouse liver. Fig 3B shows typical SPM extracted RNA quality at 1 g for both *E*. *coli* and mouse liver. Ct values from RT-qPCR runs using RNA isolated from the SPM were also similar to results from Qiagen purified RNA. Residual *E*. *coli* RNA purified in microgravity was returned to Earth on SpaceX CRS-8 for Bioanalyzer testing. The RNA extracted in microgravity (RIN 8.5) was compared to that of RNA extracted at 1 g on the same day (RIN 8) and is of comparable quality (Fig 3B) showing that RNA was successfully purified on the ISS.

#### *E*. *coli* RNA isolation and RT-qPCR analyses

After confirming that qPCR in microgravity was possible and with results comparable to 1 g controls, we proceeded to test the capability of the hardware system to isolate and process samples in microgravity. An *E*. *coli* sample, used to test the system’s capabilities with prokaryotic cells, was processed and analyzed on ISS as described previously. The RT-qPCR assays tested included singleplex (Fig 3C), duplex (Fig 3D), and triplex (Fig 3E) reactions (S1 Table), each performed in quadruplicate to improve statistical sampling. All microgravity and 1 g control reactions resulted in readable Ct data. In agreement with previous results, microgravity Ct values exhibited increased variability compared to 1 g controls. Despite the greater variability observed in microgravity Ct values, no statistically significant difference was found between the microgravity and 1 g control data. One outlier was identified in the microgravity data and was not used in statistical comparisons. Also, one microgravity triplex tube did not give data in the FAM channel (dnaK).

#### Mouse RNA isolation and RT-qPCR

The final study explored the system’s capabilities to isolate RNA from a mammalian tissue, using the resulting RNA as the template in RT-qPCR analysis. Similar to the *E*. *coli* experiment, quadruplicates of singleplex (Fig 3F), duplex (Fig 3G), and triplex (Fig 3H) RT-qPCR reactions were conducted. A significant correlation between the microgravity and 1 g control data was observed, however, microgravity data exhibited increased variability. These results indicate that RNA was successfully isolated from mouse liver tissue during spaceflight.

Singleplex and duplex assays of the reference genes gapdh and rpl19 showed no statistical differences between microgravity and 1 g control. However, in spaceflight, no gapdh-FAM signal was observed during triplex assays whilst 1 g control triplex assays resulted in detection of all three genes.

### Repeat run of *E*. *coli* RNA isolation and bubble mitigation

**Fig 4:**
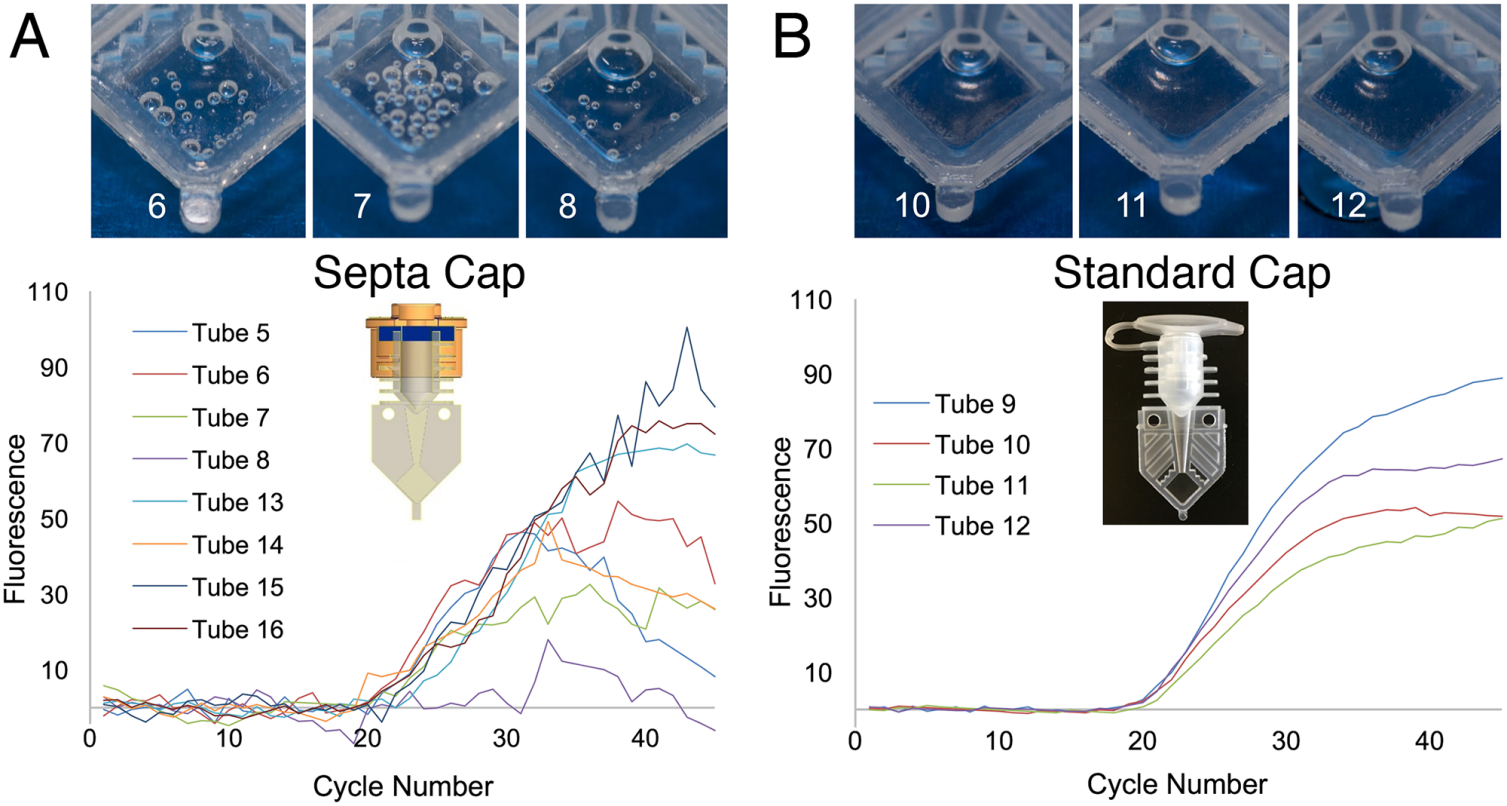
HEX amplification curves and photos from microgravity reactions comparing different caps. (A) HEX amplification curves when our modified septa caps are used. (B) HEX amplification curves from tubes sealed with the standard commercial SmartTube Caps. Post run photos of representative tubes are shown above.

Following the completion of on-orbit activities, an opportunity arose several months later to test bubble formation mitigation strategies while repeating the *E*. *coli* isolation activity. To test this, half of the modified reaction tube septa caps were replaced with standard SmartTube caps. However, the age of the sample preparation reagents was past the latest longevity testing duration and this test resulted in compromised Ct values in the FAM and Cy5 channels. The HEX channel appeared to be unaffected as seen in Fig 4. A reduction in noise was observed with the use of standard caps (Fig 4B) as compared to the typical noise seen when septa caps are used (Fig 4A). Additionally, photos taken of the tubes after completion of the RT-qPCR run show an increase in the number of bubbles in the septa capped tubes as compared to those with standard caps. We hypothesize that the internal tube pressure created when inserting the standard caps helps mitigate bubble formation. This was corroborated by sideways and inverted (upside down) ground thermocycling experiments that also trapped bubbles.

## Discussion

One of the major obstacles to space exploration going forward in the coming decades is the fact that microgravity research outposts such as ISS are so closely dependent on Earth support of everyday activities, and lack the ability to operate autonomously. The National Academies of Sciences in their Decadal Survey [5] have identified some of these limitations as important concerns and offered direction that the ISS should operate more like Earth-based laboratories, allowing for experiments to be designed, conducted, modified and iterated in space. Despite the thousands of investigations already conducted on ISS [31], there are still overwhelming limitations [32, 33] that prevent science and specifically molecular biology measurements of gene expression from being conducted and analyzed on-orbit.

From its inception, the NASA WetLab-2 project has sought to address these concerns by introducing new, time-efficient, practical and flexible molecular biology gene expression analysis capabilities to the ISS. The WetLab-2 hardware system can now rapidly perform RNA isolation on-orbit and use those RNAs to immediately conduct RT-qPCR analyses, or other novel analyses such as nanopore-based RNA sequencing. The utility of WetLab-2 can be applied to many molecular biology experimental designs using a variety of sample types and provides investigators with improved insight and interactivity during experimentation. Overall, the on-orbit process of total RNA isolation and gene expression analysis is accomplished in a span of approximately 3 h without the requirement for a highly experienced user and provides a new capability to ISS that will greatly expand the limits of possible on-orbit science.

Successful development and deployment of the WetLab-2 system to the ISS depended on solving a number of challenges such as limited refrigeration availability, hazardous reagent use allowances, power consumption, and availability of crew time. In addition, the suite of hardware and fluidic components had to function in microgravity and provide ease of use for non-expert operators.

In order to perform thermal cycling on-orbit, a suitable, commercially available thermal cycler that was capable of operating in a microgravity environment was identified. Specifically, the Cepheid SmartCycler best met the requirements without significant modifications. The instrument is modular, has reduced power requirements and offers assay wells with individual thermal protocol programming allowing for simultaneous running of assays with different optimal primer annealing temperatures. Validation testing of this instrument included physical durability tests and validation of all 16 sample sites.

TaqMan assays were selected because of the ability to multiplex assays with multiple color probes in a single tube, and because alternatives such as SYBRGreen DNA intercalating dyes are carcinogenic, resulting in safety concerns for the ISS crew and imposing elevated containment requirements.

Next we developed a RNA isolation and RT-qPCR workflow using non-hazardous reagents suitable for room temperature storage. A RNA binding column and cell lysis system for RNA purification was selected from Claremont BioSolutions for its ease of integration [21, 22]. In collaboration with Claremont, the RNA extraction protocol was modified to meet room temperature storage and low toxicity requirements. RNA extracted from the SPM proved to be of sufficient high quality and quantity for gene expression studies utilizing RT-qPCR (Fig 3B). Longevity testing of loaded SPMs showed no degradation of the reagents for at least 26 weeks (S2 Text).

We proceeded to develop a lyophilized all-in-one detection assay to minimize crew time and storage requirements. Lacking instruments to evaluate RNA purity and potential gDNA contamination, intron-spanning primers that give a product approximately 100 bases in length were used in eukaryotic samples. In prokaryotic samples, DNase enzyme [34] was included due to the lack of introns in the coding region [35]. To ensure that lyophilized assays provided similar results to those obtained using wet bench chemistry, we performed extensive validation testing. Our results indicate no significant differences between lyophilized and commercial non-lyophilized assays (see Table 2). When refrigerated, the lyophilized assays were stable with a shelf life of at least 52 weeks (S2 Text). To provide longer shelf life and room temperature storage conditions in the future, we plan to use the standard commercial caps, as they appear to perform better in initial results from ongoing long-term storage tests.

Before the WetLab-2 system could be made available to researchers, the system was validated to ensure that the data obtained on-orbit was not affected or biased by microgravity conditions, including surface tension-dominated fluid dynamics and lack of convection [36]. Specifically, because PCR consists of repeated cycles of rapid heating and cooling, lack of gravity-induced convection mixing in microgravity may limit heat transfer throughout the sample to only conduction which may require longer denaturation and annealing/extension times.

To validate PCR efficiency in microgravity, control qPCR reactions were conducted once the system was installed on-orbit. These tests included validation with a range of pre-prepared DNA template concentrations (low, mid and high) to assess PCR efficiency. Three replicate runs were conducted, of which two were successful in returning qPCR amplification curves that showed close correlation to 1 g controls. Furthermore, the microgravity Ct results were comparable to their respective 1 g controls (Fig 2 and S1 Table). Although amplification was successful, microgravity data showed increased variability within replicate sets. Analysis of the amplification plots, post-assay tube windows, and follow up experiments suggest that this increased variability may be caused by degassing bubble formation that interferes with fluorescence detection. In subsequent ground-based experiments, we found that these bubbles rise out of the tube detection window under normal 1 g conditions. Conversely, in microgravity, bubbles form, are retained and expand in the tube optical window during thermocycling, creating noise (choppiness) in the resulting amplification curves. Despite higher variability when using septa caps, our data show that qPCR can be conducted in the SmartCycler using TaqMan assays in microgravity conditions, and that resulting Ct values are comparable to those of 1 g controls, albeit noisier (Fig 2G & 2H). These results indicate that the physical alterations of fluid and thermal dynamics in microgravity are not sufficient to alter enzymatic amplification of DNA during PCR thermal cycling in the SmartCycler.

To validate that the WetLab-2 system could successfully isolate RNA from intact cells and tissue, as well as perform RT-qPCR analyses on-orbit, we prepared and froze samples of *E*. *coli* and mouse liver tissue on the ground. Results show that RNA was successfully isolated on-orbit using the SPM and that singleplex and multiplex RT-qPCR analyses from both *E*. *coli* cells and mouse liver tissues resulted in successful qPCR amplification (Fig 3 and S1 Table). Successful multiplex RT-qPCR allows for the use of ratiometric methods; expression of genes of interest can be measured relative to reference genes in the same tube, thereby effectively normalizing variability that may exist in RNA concentrations or other artifacts of microgravity PCR amplification.

Comparison of raw background fluorescence data showed significant differences between microgravity and 1 g control samples (S1 File), however, no significant differences in Ct values. In agreement with genomic DNA standards data, increased variability was also observed in the microgravity samples using SPM-derived *E*. *coli* and mouse RNA, likely due to bubble formation during thermal cycling.

As this variability in Ct values may limit the utility of the system for researchers, a follow up “volunteer science” experiment was conducted by astronaut Kate Rubins. This test was aimed at reducing or eliminating bubbles formed during thermal cycling and showed that the positive pressure formed in the tubes when closed using a standard cap drastically reduces bubbles and results in smooth amplification curves similar to those typically seen during RT-qPCR runs in 1 g (Fig 4). This run yielded data with less variability; standard deviations up to 0.659 Ct for standard caps compared to 1.035 Ct for septa caps. Therefore, for future experiments, we intend to use standard commercial tubes to suppress degassing bubble formation.

**Fig 5:**
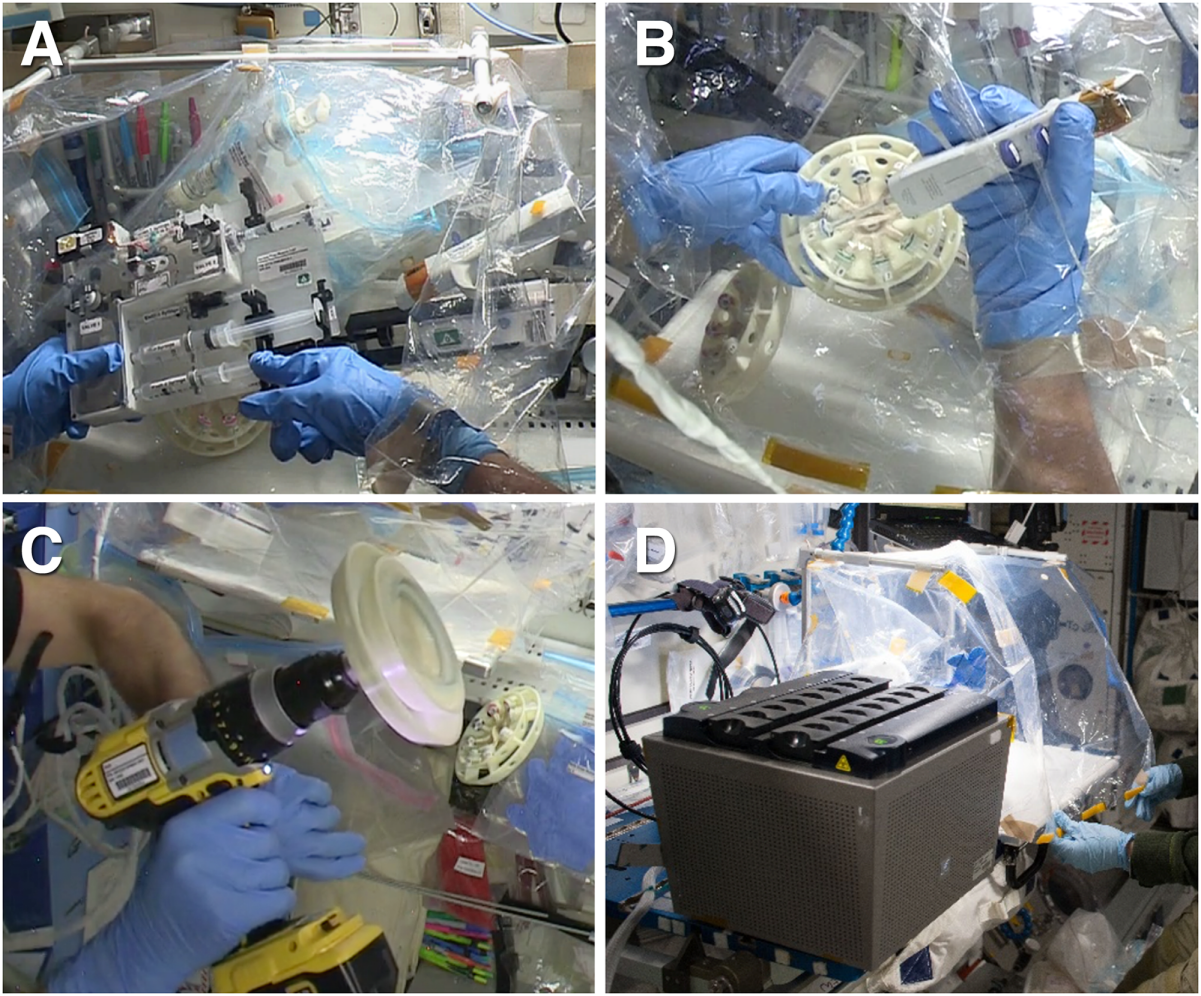
Wetlab-2 ISS operations. A) On-orbit *E*. *coli* RNA isolation using the SPM inside the DGB, B) filling tubes using the repeater pipette, C) Rotor centrifugation using the ISS cordless drill D) SmartCycler and DGB set up. Image credits: NASA.

Through these experiments, we have demonstrated that qPCR can be successfully performed in the microgravity environment of space and developed an system of molecular biology tools and reagents for extracting and quantitatively analyzing RNA from biological samples on-orbit and generating RT-qPCR Ct data (See photos of actual on-orbit operations in Fig 5).

In conclusion, the WetLab-2 ISS system is now validated and ready for future end-to-end gene expression analysis on-orbit, providing prompt results and data reporting from multiple cell and tissue biological samples. This new capability allows investigators unprecedented flexibility with ISS research and the opportunity to re-design and iterate experiments based on data received in almost real-time. The WetLab2 system is publicly available to investigators for research on ISS [37] and can also potentially be used to analyze environmental samples, for medical diagnostics, or to provide purified RNA and cDNAs for expressome sequencing and global gene expression analysis using nanopore sequencing [17] as our group recently demonstrated [38]. Overall the novel molecular biology capabilities we describe here for for ISS, although trivial on earth, are an important step forward in the quest for making possible the exploration and development of space for human use.

## Supporting information

S1 Text Detailed description of WetLab-2 system. (DOCX)

S2 Text Hardware testing methods and results. (DOCX)

S1 File Background fluorescence data. (PDF)

S1 Table Cycle threshold values and p-values for microgravity and 1 g control data. Complete Ct data from *E*. *coli* Genomic DNA validation, *E*. coli, and mouse experiments. Microgravity data are from on-orbit operations while 1 g control are from equivalent experiments run on the ground. Low, Mid, and High represent template concentrations of 0.01, 1.0, and 100 ng, respectively. Singleplex assays were amplified independently of one another, while Duplex and Triplex assays were amplified in multiplex assays, combined as shown. p-values are from t-test analyses, ^Ɨ^ denotes statistical outliers not included in t-test analyses.
(DOCX)
