# Supplementary material for: Microgravity validation of a novel system for RNA isolation and multiplex quantitative real time PCR analysis of gene expression on the International Space Station
Source: PLoS One. 2017 Sep 6;12(9):e0183480. doi: 10.1371/journal.pone.0183480 (PMC5587110; doi:10.1371/journal.pone.0183480)
Supplement: S1 Text — (DOCX) [file pone.0183480.s001.docx]

**S1 Text. Detailed description of WetLab-2 system.**

The WetLab-2 System consists of a suite of hardware modules including a Sample Transfer Tool (STT), Sample Preparation Module (SPM), Pipette Loader (PL), repeater pipette (Eppendorf, Hamburg, Germany), reaction tubes, centrifuging rotor, cordless drill, qPCR thermocycler and disposable glove bag (DGB), as shown diagrammatically in Fig 1. All modules are designed to keep manipulations simple enabling individuals with no molecular biology laboratory experience to complete the procedures with minimal training.

Experiment specific consumables launch to the ISS. At the time of sample processing, the sample is obtained using the Sample Transfer Tool. All items are loaded into the Disposable Glove Bag where the RNA is extracted using the SPM then passed through the Pipette Loader to remove air and load the Repeater Pipette. RNA is dispensed into the Reaction Tubes with the Repeater Pipette. The rotor is used to centrifuge the reaction tubes placing the RNA in the reaction window and rehydrating the lyophilized reagents. The tubes are loaded into the SmartCycler to conduct RT-qPCR analysis. Data from the SmartCycler can then be downlinked to the investigator. Alternatively, the purified RNA can be returned to the investigator for further analysis.

The STT is used to obtain, store, and input the biological sample into the SPM. It comes in two formats: a standard Luer lock syringe or a specially designed double-containment syringe (ACT^2^; Techshot, Greenville, IN) for use with hazardous samples. To ensure that the WetLab-2 System connection interface is compatible with most other ISS biological research devices, industry standard luer lock fittings are employed for fluidic inputs/outputs.

On the ISS, samples and hardware modules are gathered from stowage and loaded into the DGB, which was cleaned pre-launch to remove RNases. After loading, the DGB is hermetically sealed and all operations, up to and including dispensing RNA into the reaction tubes, are performed through gloves integrated into the DGB sidewalls. In the DGB, Reaction Tubes (Cepheid SmartTubes with modified caps) containing lyophilized components including primers and Taqman probes, are inserted onto the centrifuging rotors. The tubes are fitted with custom split-septa caps to provide fluid containment during repeater pipette dispensing in microgravity. Alternatively, standard SmartTubes can also be used.

The sample preparation procedure begins by attaching the STT to the SPM and injecting a biological sample in lysis buffer. The SPM employs a modified Claremont BioSolutions procedure to lyse the cells and purify the RNA, based on a bead-beater and capture column (Claremont BioSolutions OmniLyse and RNAexpress™ column). Sequential wash, air purge and elution steps are then performed, leaving the purified RNA in a detachable syringe. This RNA syringe is removed from the SPM and attached to the PL. The Repeater Pipette is attached to the PL and RNA is slowly introduced through a bubble trap to remove air. The RNA solution is then drawn into the repeater pipette for dispensing into the Reaction Tubes.

Tube loading is accomplished by penetrating the split-septa of the modified caps with the repeater pipette tip and dispensing 25 μl of RNA. Once all 16 tubes are loaded, the rotors are removed from the disposable glove bag and mounted onto the cordless drill. Each rotor is spun for 30 s to drive the RNA into tube reaction windows, rehydrating the lyophilized components. After rotor spinning, the tubes are loaded into a commercial thermocycler (SmartCycler; Cepheid) for RT-qPCR analysis. Thermal protocols for the SmartCycler can be uploaded by researchers on Earth for the crew to select for a given run. When the run is complete, data can be analyzed on-station, or downlinked to the ground within two hours.
