# Supplementary material for: Microgravity validation of a novel system for RNA isolation and multiplex quantitative real time PCR analysis of gene expression on the International Space Station
Source: PLoS One. 2017 Sep 6;12(9):e0183480. doi: 10.1371/journal.pone.0183480 (PMC5587110; doi:10.1371/journal.pone.0183480)
Supplement: S2 Text — (DOCX) [file pone.0183480.s002.docx]

**S2 Text. Hardware testing methods and results**

**Long-term Storage and Biocompatibility Testing**

Since liquids and reagents may potentially be stored for long periods of time, all hardware was tested for biocompatibility. The components that may be stored with reagents for long periods of time include the ACT^2^, Finger Loop Syringe, SPM, and Reaction Tubes, as summarized in the Table below. Results from this testing revealed that after 4-6 weeks, lyophilized assays exhibited significant degradation when stored at ambient temperatures. This degradation was attributed to off-gassing of moisture and/or other VOC’s from the custom cap that was manufactured with ABS-M30 using additive manufacturing (3D printing). A number of mitigation strategies were tested and implemented, including, thorough cleaning and bake outs of the cap material prior to use, inclusion of an extra desiccant pack in each pouch, and storage of the sealed pouch at 4°C. Under these conditions, assays were found to be stable for at least 52 weeks at 4°C.

**Table. Tested duration of reagents stored in hardware.**

| **Hardware Component** | **Contents** | **Conditions** | **Duration** |
| --- | --- | --- | --- |
| ACT^2^ | *E. coli* | -80°C | 28 weeks |
| Finger Loop Syringe | Mouse liver tissue | -80°C | 20 weeks |
| SPM | Wash and Elution Buffers in syringes | Ambient | 26 weeks |
| Reaction Tubes | Lyophilized assays | 4°C | 52 weeks |

All reagents performed well through the durations listed.

**Thermocycler Validation**

Cepheid SmartCycler® thermocyclers were procured for use aboard the ISS. Both the ISS and control unit were validated prior to launch to assess intra-machine variability. Validation was performed by following documented procedures for controlled assay preparation and qPCR amplification, then evaluating the accuracy and consistency of fluorescence values and Ct values generated by each module. Assays were prepared using a triplex E. coli assay including DNA template, primers and probes for dnaK-FAM, rpoA-HEX, and srlR-Cy5, GoTaq Probe Master Mix, and molecular biology grade water. Assays were stored for up to one week at 4°C and were allowed to equilibrate at room temperature for a minimum of 30 min before running. Amplification was achieved via a standard thermal protocol that included a single activation step (95°C, 2 min) and 45 cycles of denaturation (95°C, 15 s) and annealing/extension (54°C, 60 s). A total of 16 replicates were run and used to compare and validate the machines.

The Cepheid SmartCycler unit selected to be flown to the ISS was tested before launch to ensure the unit was in good working order. In addition to physical durability tests that verified tolerance to the stresses of launch and microgravity operations, a biological functional test was conducted to verify qPCR performance. This test evaluated each of the 16 sample sites across three optical channels to ensure consistent, reproducible thermal cycling and fluorescence detection. Triplex E. coli assays with FAM, HEX, and Cy5 fluorophores were performed using genomic DNA as the template. Additionally, a similar functional test was run on a ground unit for comparison and no significant differences between the two units were detected.
