# Supplementary material for: Microgravity validation of a novel system for RNA isolation and multiplex quantitative real time PCR analysis of gene expression on the International Space Station
Source: PLoS One. 2017 Sep 6;12(9):e0183480. doi: 10.1371/journal.pone.0183480 (PMC5587110; doi:10.1371/journal.pone.0183480)
Supplement: S1 File — (PDF) [file pone.0183480.s003.pdf]

S1 File. Background fluorescence data

Raw background fluorescence data was collated from cycles 1 to 10 from all genomic DNA microgravity and 1 g control runs to determine if there were differences in the background/baseline as well (Fig S3). Individual microgravity runs exhibit significantly reduced background fluorescence intensity as compared to 1 g controls, for run 1 (56.05 +/- 17.49, 90.96 +/-12.69, respectively, p<0.0001), and for run 2 (51.16 +/- 15.90, 89.08 +/- 12.54, respectively, p<0.0001). The figure also shows a clear difference in the trend of the background values from cycles 1 to 10 between microgravity and 1 g. Specifically, the microgravity background fluorescence values decrease with increasing cycle number while the 1 g control values remain flat or have a slight upward trend. Linear regression analysis revealed a significant difference between microgravity and 1 g control groups (*p*< 0.0082). These alterations may have been due to the formation of bubble in early cycles resulting in a reduction in the baseline signal.

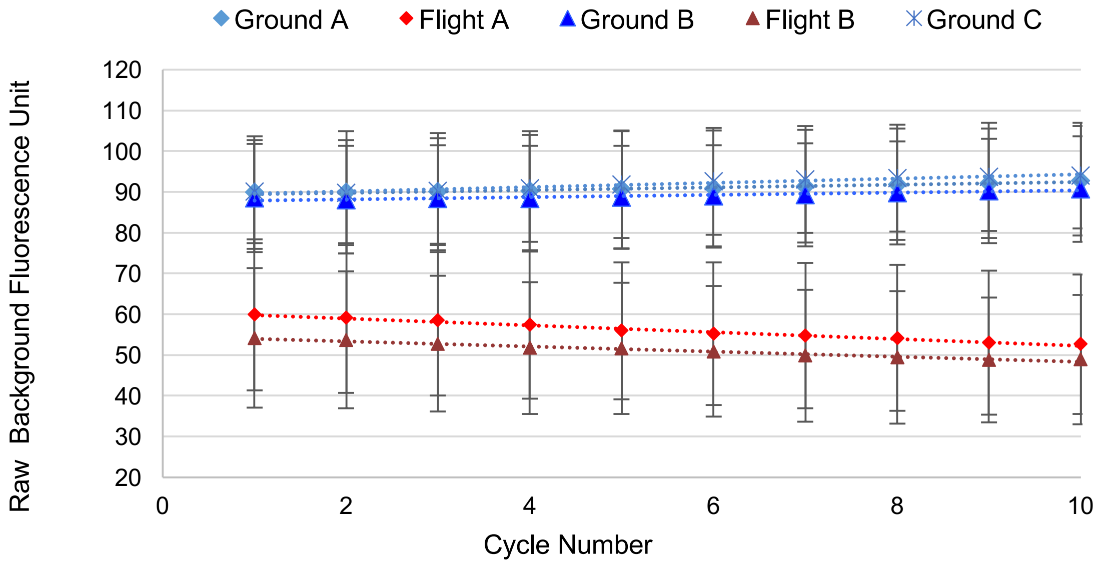

Background fluorescence from cycles 1 to 10. 1 g controls (blue) show a flat to upward slope and microgravity runs (red) show a downward slope.
