# Supplementary material for: Microgravity validation of a novel system for RNA isolation and multiplex quantitative real time PCR analysis of gene expression on the International Space Station
Source: PLoS One. 2017 Sep 6;12(9):e0183480. doi: 10.1371/journal.pone.0183480 (PMC5587110; doi:10.1371/journal.pone.0183480)
Supplement: S1 Table — Complete Ct data from E. coli Genomic DNA validation, E. coli, and mouse experiments. Microgravity data are from on-orbit operations while 1 g control are from equivalent experiments run on the ground. Low, Mid, and High represent template concentrations of 0.01, 1.0, and 100 ng, respectively. Singleplex assays were amplified independently of one another, while Duplex and Triplex assays were amplified in multiplex assays, combined as shown. p-values are from t-test analyses, Ɨ denotes statistical outliers not included in t-test analyses. (DOCX) [file pone.0183480.s004.docx]

**S4 Table. Cycle threshold values and p-values for microgravity and 1 g control data**.

|  | **Microgravity** (Ct) | | | |  | **1 g control** (Ct) | | | |  | **Microgravity** (Mean +/- SD) | | **1 g control** (Mean +/- SD) |  | p-value |
| --- | --- | --- | --- | --- | --- | --- | --- | --- | --- | --- | --- | --- | --- | --- | --- |
| ***E. coli* Genomic DNA** | | | | | | | | | | | | | | | |
| *First run* |  |  |  |  |  |  |  |  |  |  | |  |  |  |  |
| Low | 29.23 | 28.67 | 30.26 | 31.25 |  | 30.08 | 29.61 | 29.37 | 29.64 |  | | 29.85 +/- 0.99 | 29.68 +/- 0.26 |  | 0.78 |
| Mid | 24.99 | 23.51 | 23.48 | 23.76 |  | 23.83 | 23.36 | 23.21 | 23.17 |  | | 23.94 +/- 0.62 | 23.39 +/- 0.26 |  | 0.23 |
| High | 18.18 | 14.84 | 16.81 | 17.94 |  | 17.32 | 16.95 | 16.56 | 16.59 |  | | 16.94 +/- 1.32 | 16.86 +/- 0.31 |  | 0.92 |
| *Second run* |  |  |  |  |  |  |  |  |  |  | |  |  |  |  |
| Low | 29.94 | 30.96 | 30.64 | 31.19 |  | 29.86 | 29.62 | 29.8 | 29.95 |  | | 30.68 +/- 0.47 | 29.81 +/- 0.12 |  | 0.04 |
| Mid | 23.61 | 24.21 | 23.88 | 22.86 |  | 23.55 | 23.19 | 23.24 | 23.42 |  | | 23.64 +/- 0.5 | 23.35 +/- 0.14 |  | 0.39 |
| High | 18.82 | 17.51 | 18.21 | 17.93 |  | 17.24 | 16.45 | 16.81 | 16.58 |  | | 18.12 +/- 0.48 | 16.77 +/- 0.3 |  | 0.01 |
|  |  |  |  |  |  |  |  |  |  |  | |  |  |  |  |
| ***E. coli*** |  |  |  |  |  |  |  |  |  |  | |  |  |  |  |
| *Singleplex* |  |  |  |  |  |  |  |  |  |  | |  |  |  |  |
| dnaK | 19.52 | 20.92 | 20.87 | 30.45^Ɨ^ |  | 20.12 | 21.72 | 22.35 | 22.61 |  | | 22.94 +/- 4.37 | 21.7 +/- 0.97 |  | 0.14 |
| rpoA | 22.2 | 21.23 | 27.48 | 24.8 |  | 21.21 | 21.28 | 20.11 | 21.33 |  | | 23.93 +/- 2.43 | 20.98 +/- 0.51 |  | 0.12 |
| *Duplex* |  |  |  |  |  |  |  |  |  |  | |  |  |  |  |
| dnaK | 22.76 | 22.93 | 32.31 | 20.38 |  | 23.47 | 23.24 | 22.89 | 22.1 |  | | 24.6 +/- 4.57 | 22.93 +/- 0.52 |  | 0.57 |
| rpoA | 23.3 | 23.94 | 26.97 | 19.38 |  | 21.4 | 21.65 | 22.05 | 21.2 |  | | 23.4 +/- 2.7 | 21.58 +/- 0.32 |  | 0.33 |
| *Triplex* |  |  |  |  |  |  |  |  |  |  | |  |  |  |  |
| dnaK | 30.24 | 20.65 | 26.37 | 0^Ɨ^ |  | 22.95 | 20.9 | 22.2 | 19.8 |  | | 19.32 +/- 11.66 | 21.46 +/- 1.21 |  | 0.26 |
| rpoA | 24.64 | 20.25 | 24.87 | 24.96 |  | 21.82 | 20.74 | 21.3 | 19.83 |  | | 23.68 +/- 1.98 | 20.92 +/- 0.74 |  | 0.09 |
| srlR | 27.93 | 13.82 | 14.25 | 24.27 |  | 22.86 | 23.24 | 22.49 | 22.3 |  | | 20.07 +/- 6.17 | 22.72 +/- 0.36 |  | 0.51 |
|  |  |  |  |  |  |  |  |  |  |  | |  |  |  |  |
| **mouse** |  |  |  |  |  |  |  |  |  |  | |  |  |  |  |
| *Singleplex* |  |  |  |  |  |  |  |  |  |  | |  |  |  |  |
| Gapdh | 20.95 | 12.79 | 20.41 | 17.71 |  | 20.21 | 20.67 | 20.53 | 20.54 |  | | 17.97 +/- 3.23 | 20.49 +/- 0.17 |  | 0.27 |
| Rpl19 | 20.92 | 19.81 | 20.11 | 20.33 |  | 20.52 | 20.42 | 21.03 | 20.64 |  | | 20.29 +/- 0.41 | 20.65 +/- 0.23 |  | 0.24 |
| *Duplex* |  |  |  |  |  |  |  |  |  |  | |  |  |  |  |
| Gapdh | 22.6 | 22.22 | 16.52 | 25.35 |  | 22.81 | 22.67 | 22.07 | 22.36 |  | | 21.67 +/- 3.21 | 22.48 +/- 0.29 |  | 0.69 |
| Rpl19 | 20.95 | 21.92 | 20.72 | 20.82 |  | 21.06 | 20.79 | 20.23 | 20.57 |  | | 21.1 +/- 0.48 | 20.66 +/- 0.3 |  | 0.24 |
| *Triplex* |  |  |  |  |  |  |  |  |  |  | |  |  |  |  |
| Gapdh | 0 | 0 | 0 | 0 |  | 23.52 | 23.74 | 24.27 | 22.41 |  | | 0 +/- 0 | 23.49 +/- 0.68 |  | n/a |
| Rpl19 | 22.53 | 22.9 | 21.4 | 22.21 |  | 21.26 | 21.74 | 22.25 | 21.26 |  | | 22.26 +/- 0.55 | 21.63 +/- 0.41 |  | 0.17 |
| Fn1 | 30.99 | 30.84 | 29.48 | 16.98 |  | 25.61 | 28.33 | 24.99 | 25.39 |  | | 27.07 +/- 5.86 | 26.08 +/- 1.32 |  | 0.79 |

Complete Ct data from *E. coli* Genomic DNA validation, *E.* coli, and mouse experiments. Microgravity data are from on-orbit operations while 1 g control are from equivalent experiments run on the ground. Low, Mid, and High represent template concentrations of 0.01, 1.0, and 100 ng, respectively. Singleplex assays were amplified independently of one another, while Duplex and Triplex assays were amplified in multiplex assays, combined as shown. p-values are from t-test analyses, ^Ɨ^ denotes statistical outliers not included in t-test analyses.
